# Possible Association of Tremors and Dysarthria with Losartan Use: A Case Report

**DOI:** 10.7759/cureus.6374

**Published:** 2019-12-13

**Authors:** Troy J Fishman, Tigist A Degu, Liang Sun, Joshua K Salabei

**Affiliations:** 1 Internal Medicine, University of Central Florida College of Medicine, HCA North Florida Division, Gainesville, USA

**Keywords:** losartan, tremors, dysarthria, adverse effect, angiotensin receptor blocker, hypertension

## Abstract

Losartan is a common first-line antihypertensive medication particularly useful in a select patient population. Common side effects of the drug include headaches, dizziness, fatigue, nausea, vomiting, and anemia. The only well-documented detrimental side effect of losartan is angioedema/anaphylactoid reactions. Here, we present a case of a 56-year-old Caucasian male who developed tremors and dysarthria one hour after taking losartan. His symptoms were severe enough to require hospitalization and close monitoring. His symptoms later resolved without any targeted treatment. This is the first reported case of tremors and dysarthria associated with the use of losartan which could represent an entirely benign side effect or an initial sequela of a potentially detrimental side effect that warrants our attention.

## Introduction

Hypertension affects more than 25% of the world’s population and it remains one of the leading causes for visits to clinics and doctor’s offices [1-2]. Because of this, there are a plethora of antihypertensives available to physicians. First line drugs in management include angiotensin-converting enzyme inhibitors (ACEi) and angiotensin receptor blockers (ARBs), especially indicated for diabetic patients due to their renoprotective effects [3-4]. However, sometimes due to the adverse effects of these medications, as can be witnessed with other medications, their use is limited. Some common documented side effects of ACEi and ARBs include dry cough (seen mostly with ACEi use), headaches, dizziness, fatigue, nausea, vomiting, and anemia. The only well-documented potentially detrimental side effect of ACEi and ARBs is angioedema/anaphylactoid reactions [5]. In this case report, we discuss a case of a 56-year-old Caucasian male who presented with unusual tremors and dysarthria after taking losartan. His symptoms were severe and mimicked stroke-like and seizure-like symptoms requiring hospitalization and close monitoring. His symptoms later resolved without any targeted treatment. To our knowledge, this is the first reported case of tremors and dysarthria associated with the use of losartan. This may represent an entirely benign side effect or an initial sequela of a potentially detrimental side effect that warrants our attention.

## Case presentation

A 56-year-old Caucasian male with a past medical history of hypertension and gastroesophageal reflux disease (GERD) presented at our hospital emergency department (ED) complaining of uncontrollable tremors and dysarthria. His symptoms began at 04:00 on the day of presentation after he took his third dose of losartan that was recently prescribed by his primary care physician. He was on a 50 mg daily regimen. His symptoms began with upper extremity tremors that were worse on intention, later progressing to his lower extremities and limiting his ability to ambulate. He denied any loss of consciousness, facial droop, incontinence, and motor or sensory deficits. He also denied any personal or family history of seizures or transient ischemic attack (TIA).

**Figure 1:**
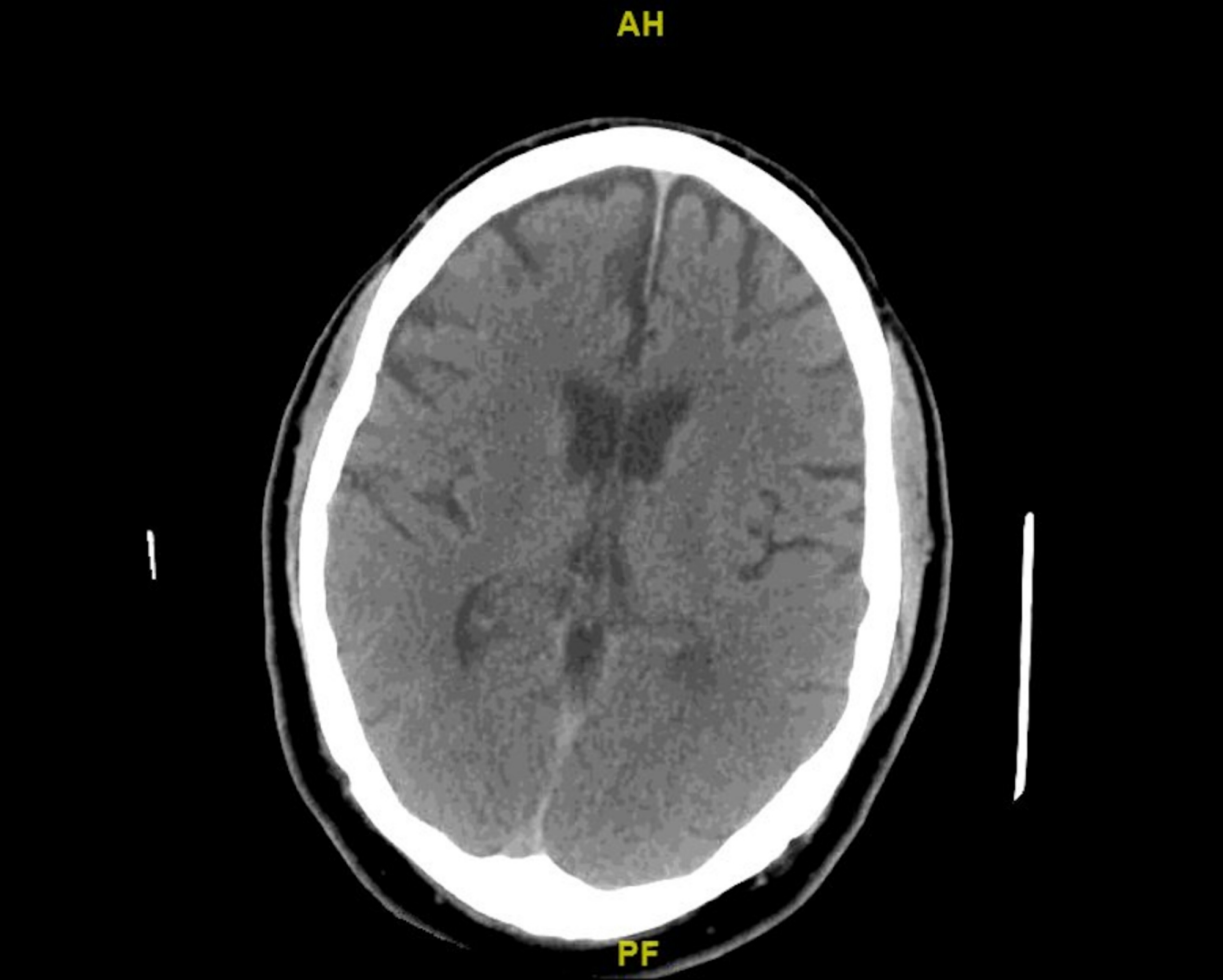
Non-contrast computed tomography (CT) scan of the brain No acute intracranial pathology can be seen in the scan.

**Figure 2:**
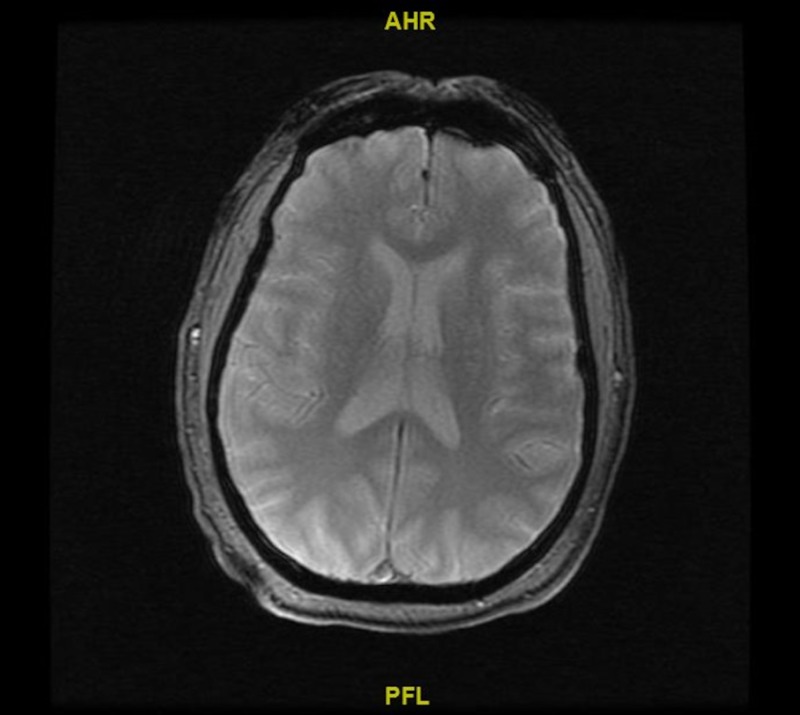
Magnetic resonance imaging (MRI) of the brain No acute intracranial pathology can be seen in the scan.

Computed tomography angiography (CTA) of the head and neck (done at a different facility prior to transfer to our facility) was unremarkable for any acute findings. He was then transferred to our facility as a stroke alert because he continued to show symptoms concerning for acute stroke (dysarthria). He was evaluated by the interventional neurology team upon arrival and stroke was ruled out based on repeat non-contrast computed tomography (CT) scan and magnetic resonance imaging (MRI) of the brain which showed no acute intracranial pathologies (Figures 1-2).

His vital signs remained stable (temperature 98.1, pulse 98, respiratory rate 16, blood pressure 140/87, and oxygen saturation 94% on room air). His labs were also unremarkable (Table 1). Abdominal ultrasound showed a fatty liver. He was put on the Clinical Institute Withdrawal Assessment for Alcohol (CIWA) protocol and then admitted for further care.

**Table 1:** Laboratory values on admission Laboratory values on admission are shown. Reference range in parenthesis.

Hemogram	Levels	Normal range
White Blood Cells	6.5	(4.5 - 11.0 thou/mm3)
Neutrophils %	63	(50.0 - 75.0 %)
Lymphocytes %	23.8	(17.0 - 42.0 %)
Monocytes %	11.9	(4.0 - 11.0 %)
Eosinophils %	0.7	(0.4 - 6.0 %)
Basophils %	0.6	(0.0 - 2.0 %)
Absolute Neutrophil Count	4.1	(thousands/mm^3^)
Red Blood Cells	4.69	(3.80 - 5.20 million cells/uL)
Hemoglobin	15.9	(12.0 - 15.0 g/dL)
Hematocrit	46.3	(35.0 - 49.0 %)
Mean Corpuscular Volume	98.6	(80.0 - 100.0 fL)
Mean Corpuscular Hemoglobin	33.9	(26.5 - 34.0 pg)
Mean Corpuscular Hemoglobin Concentration	34.4	(32.0 - 36.0 %)
Red Cell Distribution Width	14.6	(<17.0 %)
Platelet Count	132	(150 - 450 thousand/mm3)
Mean Platelet Volume	7.8	(6.6 - 10.2 fL)
Chemistry		
Sodium	136	(136 - 145 mmol/L)
Potassium	3.6	(3.5 - 5.1 mmol/L)
Chloride	101	(98 - 107 mmol/L)
Carbon Dioxide	25	(21 - 32 meq/L)
Anion Gap	13.6	(3.0 - 15.0 mEQ/L)
Blood Urea Nitrogen	7	(7 - 18 mg/dL)
Creatinine	0.83	(0.60 - 1.30 mg/dL)
Estimated Glomerular Filtration Rate (Non-African American)	116	(=>90)
Glucose	87	(74 - 106 mg/dL)
Calcium	9.2	(8.5 - 10.1 mg/dL)
Toxicology		
Alcohol	X < 3.0	(x < 3.0)

During hospitalization, he was managed symptomatically with intravenous fluids and lorazepam. He was put on neurology checks every four hours, losartan was discontinued, and he was started on amlodipine and lisinopril. Throughout his hospital course, he was administered lorazepam twice, with each dose more than 12 hours apart. His last dose of lorazepam was given at 22:45 on the day prior to discharge and he was discharged from the hospital 16 hours later without needing any more lorazepam. By the time of discharge, his tremors and dysarthria had completely resolved, and, thus, he was discharged with instructions to follow up with his primary care doctor and neurologist within a week.

## Discussion

Our patient reported routinely drinking two-three glasses of wine per night, with his last glass of wine at about 17:00 the previous day. His symptoms onset was at 04:00 the following morning (i.e., about 11 hours from his last drink). Upon arrival to the ED, he was put on the CIWA protocol, in addition to other tests and assessments done to rule out any intracranial pathology. Throughout his hospital course, the patient was administered lorazepam twice, with each dose more than 12 hours apart. Therefore, it is unlikely that his symptoms were caused by alcohol withdrawals because patients actively withdrawing from alcohol typically need more frequent doses of benzodiazepines [6]. More so, he started experiencing new-onset tremors within one hour after taking his third dose of losartan. In addition to this, the patient and wife reported that they often abstain from alcohol for two-three days and neither of them had ever experienced any signs of withdrawals.

The patient’s symptoms were unlikely to be caused by an acute intracranial process, given the negative findings in imaging studies. Also, his physical exam findings did not show any motor or sensory deficits, again confirming the unlikeliness of an intracranial pathology. His white blood cells (WBC) and red blood cells (RBC) were within normal limits and he did not present with any fever. His urine toxicology screen was negative for other recreational drugs, and his blood ammonia and lactic acid levels were within normal limits, again confirming that other causes, other than the use of losartan, were unlikely. The patient also denied any family history of tremors and dysarthria. A reconciliation of his home medications showed that he was on a 40 mg daily pantoprazole regimen for GERD. He remained stable throughout hospitalization and his symptoms resolved without any medical intervention before his discharge from the hospital. His prescription was changed to a different first-line antihypertensive belonging to a different class.

An online search for tremor and dysarthria as possible side effects of losartan yielded only two websites which mentioned “shakiness” and “slurred speech” as side effects of the drug (https://www.mayoclinic.org. and https://www.drugs.com). However, these side effects had not been mentioned on UpToDate, Medscape, or Epocrates. Although we are not fully knowledgeable about how these side effects are reported on these websites, it is evident that, at least, these effects have been previously experienced, though not properly documented. We are, therefore, not sure about the outcomes of the patients who experienced these side effects, reiterating the need for careful documentation so as not to miss potential deleterious drug side effects.

## Conclusions

In conclusion, we report a case of new-onset tremors and dysarthria, most likely associated with the use of losartan. Since the patient’s symptoms resolved without any specific intervention, this may represent a completely benign side effect. As this is the first documented report of such an effect associated with losartan use, it is likely that this is a very rare phenomenon that affects the neurological and/or musculoskeletal systems given that the drug has been in circulation in the United States of America since 1995. At this point, it is not clear if the effects witnessed by this patient were solely due to losartan or they were due to an interaction of losartan and pantoprazole. Thus, it would be difficult to draw any definitive conclusions until more publications of similar events associated with losartan usage are written.
